# Tracing the Phonetic Space of Prosodic Focus Marking

**DOI:** 10.3389/frai.2022.842546

**Published:** 2022-05-19

**Authors:** Simon Roessig, Bodo Winter, Doris Mücke

**Affiliations:** ^1^IfL-Phonetik, University of Cologne, Cologne, Germany; ^2^Department of English Language and Linguistics, University of Birmingham, Birmingham, United Kingdom

**Keywords:** prosody, focus, speech production, intonation, information structure

## Abstract

Focus is known to be expressed by a wide range of phonetic cues but only a few studies have explicitly compared different phonetic variables within the same experiment. Therefore, we presented results from an analysis of 19 phonetic variables conducted on a data set of the German language that comprises the opposition of unaccented (background) vs. accented (in focus), as well as different focus types with the nuclear accent on the same syllable (broad, narrow, and contrastive focus). The phonetic variables are measures of the acoustic and articulographic signals of a target syllable. Overall, our results provide the highest number of reliable effects and largest effect sizes for accentuation (unaccented vs. accented), while the differentiation of focus types with accented target syllables (broad, narrow, and contrastive focus) are more subtle. The most important phonetic variables across all conditions are measures of the fundamental frequency. The articulatory variables and their corresponding acoustic formants reveal lower tongue positions for both vowels /o, a/, and larger lip openings for the vowel /a/ under increased prosodic prominence with the strongest effects for accentuation. While duration exhibits consistent mid-ranked results for both accentuation and the differentiation of focus types, measures related to intensity are particularly important for accentuation. Furthermore, voice quality and spectral tilt are affected by accentuation but also in the differentiation of focus types. Our results confirm that focus is realized via multiple phonetic cues. Additionally, the present analysis allows a comparison of the relative importance of different measures to better understand the phonetic space of focus marking.

## Introduction

Prosody, the rhythmic and tonal organization of speech, plays an integral role in communication. In West-Germanic languages, such as German and English, prosody is essential in signaling which parts of the utterance are in focus, and which are in the background (Lambrecht, 1994). The background domain encompasses information that “anchors” the sentence to the listener's knowledge and beliefs (the listener's “mental world”) while the focus domain contributes significant information to this background (Vallduví and Engdahl, 1996, p. 461). In other words, the focus domain contains information that the speaker believes to be significant in the discourse context; the background constituent contains information that is given or expected (Vallduví and Engdahl, 1996; Gussenhoven, 2004). Differentiating background from focus helps a speaker package information. This packaging reflects the speaker's belief about the listener's “mental model of the current conversation” (Vallduví and Engdahl, 2013, p. 19) and how the sentence fits this model.

In West-Germanic languages, the main prominence of the sentence, the nuclear pitch accent, is usually placed on the focused part. Consequently, the background part does not receive the nuclear pitch accent and is often completely unaccented—in particular, when it is post-focal, i.e., when it follows rather than precedes the focused part (post-focal deaccentuation). Examples (1) and (2) show the modulation of the nuclear pitch accent position as a function of focus structure. In the answer in (1), the subject “Nora” is in focus (indicated by subscript F for ‘focus') while “wants to meet Mary” is in the background (indicated by subscript B for ‘background'). In the answer in (2), the object “Mary” is in focus and receives the nuclear accent, while “Nora wants to meet” is in the background. In both examples, the focus structure of the answer is conditioned by the question. The location of the nuclear pitch accent is marked by bold capitals in the answers.

(1)
Q: Who wants to meet Mary?
A: [**NO**ra]_F_ [wants to meet Mary.]_B_

(2)
Q: Who does Nora want to meet?
A: [Nora wants to meet]_B_ [**MA**ry.]_F_

Both (1) and (2) are examples in which one word occurs in narrow focus (Ladd, 1980). A narrow focus can also occur when an expression is a correction of an alternative and is then referred to as contrastive focus (or corrective focus, cf. Gussenhoven, 2007), see example (3).

(3)
Q: Does Nora want to meet Jane?
A: [Nora wants to meet]_B_ [**MA**ry.]_F_

A focus structure with a domain that encompasses multiple constituents of the sentence is referred to as broad focus. A classic example illustrating broad focus is an all-new sentence as shown in the answer of example (4) where the entire answer is in focus. It has to be noted that a structure like (5) also classifies as broad focus (Lambrecht, 1994, 2000) where the subject is given and represents a topic. In both cases, the last argument receives the nuclear pitch accent by focus projection (Büring, 2003; Ladd, 2008) and is called the focus exponent.

(4)
Q: What's new?
A: [Nora wants to meet **MA**ry]_F_

(5)
Q: What's new about Nora?
A: She [wants to meet **MA**ry]_F_

These examples show that the placement of the nuclear pitch accent is crucial in determining the focus structure of the sentence or, in other words, that the focus structure determines the position of the nuclear pitch accent (Ladd, 2008). The word “Mary” occurring in focus (either in narrow/contrastive focus or as the focus exponent of a broad focus) can be expected to be phonetically different from the word “Mary” occurring in the background as in example (1). This phonetic differentiation involves prosodic modulations of the various parameters of speech production, including F0, intensity and duration, voice quality, and supra-laryngeal articulation. It also matters what type of focus is used, i.e., whether the word is in broad, narrow, or contrastive focus (Breen et al., 2010; Mücke and Grice, 2014; Grice et al., 2017; Krivokapić et al., 2017). Broadly speaking, it has been found that speakers enhance prosodic prominence gradually from broad to contrastive focus with narrow focus ranging in between the two.

There is a large and growing body of studies on various phonetic parameters in production and perception and how they relate to prosodic prominence (e.g., Baumann and Winter, 2018). However, only few studies look at the relative impact of focus realization on different phonetic parameters. Moreover, even fewer studies compare acoustic factors with articulatory ones. The present study investigates a whole suite of phonetic variables, both acoustic and articulatory, with respect to unaccented vs. accented syllables (background vs. broad), as well as with respect to different focus types (broad vs. narrow vs. contrastive). It uses an analysis layout that allows for the comparison of effect sizes and thus helps to trace the relative importance of the phonetic variables in the prosodic marking of information structure. In the next section, we will review the research literature on acoustic and articulatory cues to accentuation and beyond.

## Background

### Fundamental Frequency

Fundamental frequency (F0), which determines perceptual pitch, is undoubtedly a dominant phonetic parameter for the expression of focus. As opposed to unaccented syllables, pitch accented syllables are very often characterized by a higher F0 or more rapid changes in F0. For example, for English, Cooper et al. (1985), Eady and Cooper (1986), and Fowler (1995) found higher F0 in accented syllables in contrastive or narrow focus compared to unaccented syllables in the background. These findings are supported by Breen et al. (2010) who compared unaccented (background) to accented syllables in broad focus.

Intonation contours often present complex structures in which a simple, linear relationship of F0 to prominence (e.g., higher F0 ~ more prominence) may not be given. According to the widespread autosegmental-metrical framework (AM), pitch accents represent tonal targets with F0 being interpolated through unaccented syllables (Ladd, 2008). When F0 reaches a local maximum on an (L+)H^*^ pitch accented syllable, it is lower in the preceding and following unaccented syllables. In these cases, prosodic prominence falls together with higher F0 values (and the relation “higher F0 ~ more prominence” holds). However, pitch accents can also be falling (e.g., H+L^*^ or H+!H^*^) in which case the F0 of the accented syllable is lower than that of the preceding unaccented syllables. Consequently, for a quantification of F0 to be informative, qualitative properties of the intonation contour have to be taken into account, not just F0 height.

Regarding the differentiation of focus types by F0, several researchers found that certain F0 patterns are more common for certain focus types. Pierrehumbert and Hirschberg (1990), as well as Ito et al. (2004), describe L+H^*^, a high accent with a steep rise, as the most common accent type in contrastive focus in English. Breen et al. (2010), also for English, reported higher F0 values in accented syllables in narrow compared to broad focus. Unexpectedly, they found lower F0 values in contrastive focus compared to narrow focus. Baumann et al. (2007) in a study on German reported increasing proportions of upstepped ^∧^H^*^ and decreasing proportions of downstepped !H^*^ nuclear accents from broad to narrow focus and from narrow to contrastive focus. Upstep of an H^*^ accent means the accent is higher than the preceding H^*^ accent, while downstep signifies that the accent is substantially lower than the preceding H^*^. By using this classification, the authors concentrate on the pitch accent height relative to preceding accents, thereby emphasizing the importance of the relationship between multiple F0 targets.

Grice et al. (2017) identified a comparable pattern in their German data: the broad focus is most frequently expressed by falling accents (H+!H^*^ or H+L^*^) and contrastive focus by steep rising accents (L+H^*^). Narrow focus exhibits many L+H^*^ accents but also a substantial proportion of shallower H^*^ accents. The authors found that speakers employ considerably divergent strategies regarding their pitch accent type distributions in different focus types. While some speakers clearly produced falling accents in broad focus, steep rising accents in contrastive focus, and intermediate patterns in narrow focus, other speakers employ one accent type primarily in all three (or at least two) focus types.

A closer inspection of phonetic parameters in Grice et al. (2017) revealed striking similarities between the productions of all speakers. The first parameter, tonal onglide, quantifies the F0 distance in terms of semitones between the starred tone target (e.g., H^*^ of L+H^*^ or L^*^ of H+L^*^) and a point 30 ms before the onset of the accented syllable.^1^ In this way, the tonal onglide indicates the direction and the magnitude of the tonal movement at the same time. The sign of the onglide denotes the direction: negative values indicate falling accents; positive values indicate rising accents. The magnitude indicates how much the accentual movement falls or rises.

The second parameter, peak alignment, indicates the time lag between the onset of the accented syllable and the H tone component of the syllable. This H can be the starred tone (as in H^*^) or a leading tone (as in H+L^*^). Positive alignment values indicate that the peak is in the syllable, negative values indicate that it is before the syllable. The numerical value of the peak alignment parameter reveals how early or late the alignment is. Both onglide and alignment increase from broad to contrastive focus with intermediate values for narrow focus (broad focus < narrow focus < contrastive focus). Crucially, some speakers start with falling / early peak accents in broad focus and move to rising / later accents in narrow focus and even more rising / even later accents in contrastive focus. Other speakers start with rising onglides and alignments after the syllable boundary but adjust their values in the same direction: increasing onglide and alignment values for narrow and contrastive focus.

Regarding the perceptual side of the relation between F0 and prosodic prominence, it has been shown that F0 is important for the detection of phrasal prominence. F0 height is used by listeners to determine prominence (Baumann and Winter, 2018; Cole et al., 2019; Bishop et al., 2020) and even small F0 excursions can have a large effect on prominence ratings (Rietveld and Gussenhoven, 1985). The preference for L+H^*^ over H^*^ in contrastive focus, two accent types that often differ in terms of F0 excursion but also peak alignment, was confirmed in an eye-tracking study by Watson et al. (2008) for English. Rump and Collier (1996) provided evidence for Dutch that listeners are able to distinguish broad, narrow, and contrastive focus based on F0 contours alone. In this last study, the peak height in relation to the previous peak played an important role which is congruent with the production results of Baumann et al. (2007) for German.

### Duration and Intensity

Another important phonetic parameter of prosodic prominence is duration. Early experiments by Fry (1955), Tiffany (1959), and Lieberman (1960) revealed that stressed syllables (or vowels in stressed syllables) are longer than unstressed syllables (or vowels in unstressed syllables) in English. Although these experiments concentrate on lexical stress, it is reasonable to assume from the reported speech materials that at least some of the stressed syllables also received a pitch accent (although not necessarily a nuclear accent). Cooper et al. (1985) and Eady and Cooper (1986) provided evidence for longer durations of words that receive a pitch accent compared to unaccented words. The authors used speech materials in which the unaccented syllables were elicited in the background while the accented syllables were elicited in narrow (Cooper et al., 1985) or contrastive focus (Eady and Cooper, 1986). Breen et al. (2010) for English and Mücke and Grice (2014) for German found longer word durations in unaccented versus accented entities comparing background to broad focus. Longer durations of accented (narrow focus) compared to unaccented words (background) are also shown by Arnhold and Kyröläinen (2017) for Finnish. Avesani et al. (2007) reported similar results for Italian unaccented vs. accented syllables and vowels. Baumann et al. (2007), and Mücke and Grice (2014) for German, and Breen et al. (2010) for English demonstrated that not only the presence or absence of accentuation influences durations, but also the focus type, with durations increasing from broad to narrow focus, and from narrow to contrastive focus (syllable and foot: Baumann et al., 2007 as well as Mücke and Grice, 2014; word: Breen et al., 2010). Duration also plays a major role in the perception of prominence, as has been shown by Turk and Sawusch (1996), Cole et al. (2010), Baumann and Winter (2018), Cole et al. (2019), and Bishop et al. (2020), with longer durations leading to higher prominence ratings of words.

Furthermore, quantities determining perceptual loudness have been shown to greatly influence the perception of prominence. A very common and straightforward measure is RMS amplitude. Another widespread measure is sound pressure level, often referred to as intensity. Both are related to the power of the sound signal—RMS amplitude representing the square of power and intensity relating the power to the human auditory threshold. Fry (1955) and Lieberman (1960) provided evidence for higher intensities and amplitudes of stressed syllables in English. More specifically regarding pitch accentuation, Fowler (1995), Harrington et al. (2000), and Breen et al. (2010) reported higher intensities and amplitudes for accented compared to unaccented words in English. In Harrington et al. (2000), the category “accented” contains accented words from different focus types. In contrast, Fowler (1995) compared unaccented (background) to contrastively focused accented words. Breen et al. (2010) found higher intensities for accented syllables comparing syllables in the background vs. broad focus. In addition, the authors reported gradually increasing intensities from broad to narrow focus and from narrow to contrastive focus—demonstrating that intensity is not only used to mark accent but also to enhance prosodic prominence beyond accentuation. Similar to duration, amplitude and intensity have also been shown to be relevant acoustic cues to prominence in perception (Turk and Sawusch, 1996; Kochanski et al., 2005; Cole et al., 2010; Baumann and Winter, 2018; Bishop et al., 2020).

### Spectral Slope and Voice Quality

In addition to F0, duration, and amplitude, prosodic prominence affects the spectral slope (or spectral balance), i.e., the relation between intensities of the frequency bands in the spectrum. Sluijter and van Heuven (1996) reported for Dutch that accentuation leads to a flatter spectral slope. This result is in line with the findings of Campbell and Beckman (1997) and Sluijter et al. (1995) for English. A flatter spectral slope means that the intensity of higher frequency bands is increased relative to lower frequency bands. Listeners associate flatter spectral slopes with higher perceived prominence, as shown for English by Cole et al. (2010) and for German by El Zarka et al. (2017) and Baumann and Winter (2018). There are various ways of quantifying the spectral slope that take different frequency bands into account. A commonly used measure is H1-A3, the difference between the intensity of the first harmonic H1 (which corresponds to the F0) and the highest amplitude in the vicinity of the third formant A3 (Sluijter et al., 1995; Baumann and Winter, 2018). Another measure, spectral emphasis, represents the difference between the energy of the whole signal and the energy of a low-pass filtered signal (Traunmüller, 1997; Traunmüller and Eriksson, 2000).

Tightly connected to the spectral slope is the notion of voice quality, also known as phonation quality. According to Ladefoged's (1980) model, voice quality varies on a continuum of glottal constriction from open to closed. Toward the “open” end of this continuum, we find a breathy voice, whereas a creaky voice is found on the “closed” end of the continuum. Modal voice is located in between these two voice qualities (Keating and Esposito, 2007). The aforementioned parameter H1-A3 has proven to be a useful measure reflecting perceptually important portions of the speech spectrum and differentiating breathy from non-breathy voice (DiCanio, 2009; Garellek, 2019). Another very common measure used to characterize speech signals regarding the voice quality continuum is H1-H2. This measure represents the difference between the intensity of the first harmonic (H1) and the second harmonic (H2) in the spectrum. H1-H2 relates to the time proportion of the glottal cycle in which the glottis is open and is therefore also called the acoustic opening quotient (Lintfert, 2010; Mooshammer, 2010). To relate H1-H2 to the voice quality continuum: higher values (more open) are measured for breathy voice; lower values (less open) are measured for creaky voice; the values of modal voice are located in between.

Regarding the relation between prominence and H1-H2, the literature provides differing results. Sluijter et al. (1995) reported that in English, H1-H2 is lower for accented as opposed to unaccented syllables. This result is in line with Epstein (2003), who showed for English that accentuation leads to a tenser voice with smaller opening proportions in the glottal cycle. In contrast to this, Campbell and Beckman (1997) found higher H1-H2 values for pitch accented syllables in English, and Mooshammer (2010) did not identify any effect of accentuation on H1-H2 in her German data (although she found higher values for stressed vs. unstressed syllables). Interestingly, both lower and higher H1-H2 values in accented vs. unaccented syllables seem to be plausible: Speakers may use modal voice in accented syllables but tend toward the breathy end of the continuum in unaccented syllables. Likewise, speakers tend to use creaky voice at the end of utterances with low F0. Consequently, syllables in later positions in the phrase are often characterized by creaky voice. While voice quality is best described as an effect of finality here, there may also be an interaction with prosodic prominence: Since post-focally unaccented syllables are often produced with low F0, creaky voice may occur more frequently in unaccented syllables than in accented ones (which have higher F0) even if the position in the phrase is held constant. An additional complicating factor for the interpretation of differing H1-H2 results is that the amplitudes of harmonics are affected by the vowel formants whose influence is not always taken into account (see Iseli et al., 2007 for a correction method).

Empirical evaluation of H1-H2 has shown that it is affected by several factors, including nasality and F0 (Garellek and Keating, 2011; DiCanio, 2012; Simpson, 2012). Therefore, H1-H2 is best interpreted in relation to other voice quality parameters. Garellek (2019) sketches an addition to the model of Ladefoged (1980) mentioned above by including a quantification of the inharmonic spectral components as an orthogonal dimension to H1-H2. A well-suited parameter here is the harmonics-to-noise ratio (HNR) that characterizes the relation between the source spectrum's harmonic and inharmonic components. In this two-dimensional model with H1-H2 and HNR, modal voice is still located in-between creaky voice (lower) and breathy voice (higher) on the H1-H2 dimension. In addition, modal voice is characterized by higher HNR values compared to both creaky and breathy voices. Creaky voice exhibits lower values for both H1-H2 and HNR than modal voice, whereas breathy voice has higher H1-H2 and lower HNR than model voice.

### Supra-Laryngeal Articulation and Formant Frequencies

Importantly, prosodic prominence has been shown to influence supra-laryngeal articulation as well. That is, the tongue, jaw, and lip gestures for the production of consonants and vowels are affected by whether a syllable receives a pitch accent or not. Generally, it has been found that jaw movements for the opening of a vowel are larger, longer, and faster in accented syllables compared to unaccented syllables (Kent and Netsell, 1971; Kelso et al., 1985; Edwards et al., 1991; Beckman et al., 1992; de Jong et al., 1993; de Jong, 1995; Fowler, 1995; Harrington et al., 1995, 2000; Cho, 2005).^2^ It has also been reported that the lips are opened wider for vowels in accented syllables and that the movements of the lips become faster and longer (for English: Cho, 2006; for Italian: Avesani et al., 2007). The congruence between jaw and lip kinematics under prominence can be attributed to the fact that movements of the lips are largely linked to the movement of the jaw. Larger lip and jaw openings have been suggested to enhance the sonority of the vowel. In addition, more extreme tongue positions during the articulation of vowels have been reported and linked to the idea of the enhancement of place features, such as [+back]. For instance, Mooshammer and Geng (2008) found retracted tongue positions for back vowels and lower tongue positions for low vowels in pitch accented syllables in their German data. de Jong et al. (1993) showed for English that the tongue body is more retracted in the back vowel /℧/ when it occurs in an accented syllable. Cho (2005) and Kent and Netsell (1971), also for English, reported that the tongue position is shifted forward in accented front vowel /i/. Cho (2005) found a lower tongue body position in the low back vowel /α/. While the modification is often in the peripheral directions of the vowel space to enhance the place features of the vowel, the opposite direction is possible. This may be caused by an interaction with the jaw articulation that is involved in larger openings of the vocal tract: in Cho (2005), for instance, the tongue position for /i/ was lower (in addition to being fronter).

The adjustments in the supra-laryngeal articulation of vowels result in modified formant frequencies, mostly less centralized (Tiffany, 1959; Mooshammer and Geng, 2008). In particular, increased F1 values are described for the vowel /a/ indicating that the position of the tongue is lower (Harrington et al., 2000; Cho, 2005; El Zarka et al., 2017); increased F2 (and F3) values for /i/ were measured, which indicate fronting (Harrington et al., 2000; Cho, 2005). For this high vowel, however, higher F1 values have been reported when it is accented (Cho, 2005)—a result that points toward a lower tongue position and is not in line with the idea that vowels are less centralized. Again, as explained above, this may be linked to the general tendency to lower the jaw in prominent positions. So, while tongue positions and vowel formants show the general trend to be more peripheral (or hyperarticulated) when accented, lowering of the jaw to increase sonority may produce a counter tendency for high vowels.

Regarding the differentiation of nuclear-accented words in different focus types, the German data of Mücke and Grice (2014) revealed consistently larger, longer, and faster vocalic lip opening movements in broad as opposed to contrastive focus with narrow focus ranking in between the two. The differences between broad and narrow focus are not as consistent as between broad and contrastive focus. The same is true for the differences between narrow and contrastive focus. Crucially, differences between the accented focus types (broad, narrow, contrastive) appear to be larger and more consistent than differences between background (unaccented) and broad focus. Krivokapić et al. (2017) presented comparable results for English with longer gesture durations as well as longer acceleration gestural phrases in contrastive focus compared to broad focus. Again, narrow focus is not always consistently different from broad and contrastive focus: While one of the two speakers in the study shows longer lip gestures in narrow than in broad focus and also longer lip gestures in contrastive than in narrow focus, the other speaker in the study groups broad and narrow focus together and distinguishes the two from contrastive focus.

To summarize, the studies reviewed here demonstrate that prosodic prominence manifests itself in many dimensions of the phonetic signal. This applies to both the opposition of unaccented vs. accented, as well as the more fine-grained differentiation of accented words in various focus structures. The present investigation includes a total of 19 phonetic variables taking the opposition unaccented vs. accented as well as the differentiation of accented focus types into account, while at the same time looking at both acoustic and articulatory factors. The analysis approach of the current study allows a comparison of effect sizes of the variables and thereby contributes new insights into the phonetic space of accentuation and focus marking.

## Methods

### Participants and Recordings

The data set analyzed in this study consists of recordings from 27 native speakers of German producing sentences with varying focus structures.^3^ The speakers were aged between 19 and 35 at the time of recording, of whom 17 were self-identified as women and 10 as men. All speakers lived in the Cologne area and were raised in the Ripuarian dialect region around the city of Cologne (south of the Benrath line). None of the subjects had previously received special training in phonetics, phonology, or prosody, or reported any speech or hearing impairments. The subjects received compensation for their participation in the study.

The acoustic speech signals were recorded using a head-mounted AKG C520 condenser headset microphone (AKG, Stamford, Connecticut, USA) into a computer via a PreSonus AudioBox 22 VSL interface (PreSonus, Baton Rouge, Louisiana, USA) at a sampling rate of 44.1 kHz and a bit depth of 16 bit. In addition, the articulatory movements of the lips and tongue were tracked with a Carstens AG501 Electromagnetic Articulograph (EMA) (Carstens, Bovenden, Germany) simultaneously with the acoustic recordings. Sensors were placed on the upper and lower lip, tongue tip, tongue blade, and tongue body. Additional reference sensors on the bridge of the nose and behind the ears were used to compensate for head movements. The occlusal plane was rotated based on a bite-plate measure. The EMA data were recorded at 1,250 Hz, downsampled to 250 Hz, and smoothed with a 3-step floating mean. To capture the kinematic properties of the vowel production, data from the lip sensors and the backmost tongue sensor (tongue body) were analyzed. The actual recording session after the participant had been prepared lasted about 45 min including a training session.

### Procedure and Speech Materials

During the recording sessions, the participants were seated in front of a screen. They were involved in an interactive animated game that revolved around two robots working in a factory. The participants were told that one robot moves tools around the factory and the other slightly older and technologically outdated robot needs the participant's help to retrieve these tools. At the beginning of each trial, the participant saw the first robot placing the tool on an object in the factory and leaving the scene. Next, the second robot appeared at the edge of the screen, stopped before the closed factory door, and asked a question about the action of the first robot. The participant gave the answer, the door opened, the second robot entered the factory, took the tool, and left the scene.

The robot's questions were recordings of a single male native speaker of German (a trained phonetician). These questions triggered the focus structures of the participants' answers. The answers were of the form “er hat die X auf die Y gelegt” (English: “he placed the X on the Y”), e.g., “er hat den Hammer auf die Nahle gelegt.” The target word occurred in position Y denoting a fictitious object, while the word in X denoted a tool. The target word was in broad focus, narrow focus, contrastive focus, or in the background (with a contrastive focus on the tool in position X). Examples for a question-answer pair are given below: (6) for broad focus, (7) for narrow focus, (8) for contrastive focus, and (9) for background.

(6)
Q: Was hat er gemacht?
*What did he do?*
A: Er hat [den Hammer auf die Nahle gelegt]_F_.
*He put the hammer on the Nahle*.

(7)
Q: Wo hat er den Hammer hingelegt?
*Where did he put the hammer?*
A: Er hat den Hammer [auf die Nahle]_F_ gelegt.
*He put the hammer on the Nahle*.

(8)
Q: Hat er den Hammer auf die Wohse gelegt?
*Did he put the hammer on the Wohse?*
A: Er hat den Hammer auf [die Nahle]_F_ gelegt.
*He put the hammer on the Nahle*.

(9)
Q: Hat er die Säge auf die Nahle gelegt?
*Did he put the hammer on the Nahle?*
A: Er hat [den Hammer]_F_ auf die Nahle gelegt.
*He put the hammer on the Nahle*.

The questions were presented auditorily with the robot moving its mouth in synchrony. They were additionally illustrated in a thought bubble above the head of the robot.^4^ The answers that the participant had to produce were given in written form at the bottom of the screen. Many participants reported that they were able to give the answers without reading them on the screen after some trials. The participants were instructed to produce the answer with the same syntactic structure without adding words like “no.” None of the participants had any problems with this restriction. Likewise, none of the participants reported that they found the sentences unnatural or difficult.

The target words were 20 German-sounding disyllabic nonce words with a C1V1:C2? structure with stress on the first syllable. The stressed vowel V1 was either /a:/ or /o:/, while the second vowel always schwa. The first consonant was from the labial and alveolar set of /n m b l v/, the second consonant was from /n m z l v/. Each first consonant occurred twice with /a/ and twice with /o/. Each second consonant occurred four times in the whole set. The target words were pronounced as expected by all participants. A full list of the target words is given in Appendix Table A1.

Each target word was associated with a fictitious visual object. In the preparation phase, the participants were presented with all objects and target words and read the words aloud with the determiner “die” (“die Nohme” /di: ^|^no:mǝ/, “die Lahse” /di: ^|^ la:zǝ /, etc.). Each target word was produced in all four focus conditions (background, broad focus, narrow focus, contrastive focus) without repetition. A total of 80 utterances per speaker were recorded. 16 trials with different target words preceded the actual experiment session.

The trial order was randomized for each participant with the following constraints. First, subsequent trials were not allowed to contain the same target word (prepositional object, position Y in the scheme above) or tool (direct object, position X in the scheme above). Second, only 15% of the trials were allowed to have the same focus condition in the following trial. Finally, three subsequent trials with the same focus condition were not allowed.

All visual elements were drawn by a professional book illustrator. The game was developed as an animated browser app. During the recording session, the experimenter sat behind the participant and controlled the flow of the trials with a keyboard. Between trials, a pause of 4 seconds was included to make sure that the focus structure of the target sentence made reference to the current trial only.

### Annotations

Two trained annotators (one of them the first author) marked the boundaries of the stressed syllable of the target word in emuR (Winkelmann et al., 2021) and the boundaries of the vowel of the stressed syllable in Praat (Boersma and Weenink, 2001). In addition, the data were forced aligned using the Montreal Forced Aligner (McAuliffe et al., 2017) at a later stage. From the forced aligned labels, only the beginning of the determiner “die” preceding the target word was used.

The following annotation scheme was used for the intonation contour on accented target words. The annotators judged perceptually whether the nuclear pitch accent was falling or rising. Next, the annotators identified the beginning and the end of the tonal movement manually within a window of three syllables, namely, the accented syllable in the center, the preceding syllable, and the following syllable. For rising accents, the local minimum at the rising movement was annotated in the pre-accentual syllable or the accentual syllable. The local maximum at the end of the rise was labeled in the accentual syllable or the post-accentual syllable. For falling accents, the high start of the fall was labeled in the pre-accentual syllable or the accentual syllable. All utterances end with a low boundary tone. Hence, the end of the fall in the accentual or post-accentual is often hard to determine. Therefore, the midpoint of the vowel of the accented syllable was marked as the end of the fall. Finally, the low boundary tone at the end of the utterance was marked.

### Measurements

The measurements described in the following were performed with Praat and Parselmouth (Jadoul et al., 2018) as a Python interface to Praat. First, we measured the duration (in ms) and RMS amplitude (in Pa) of the stressed vowel of the target word. In addition, we extracted mean values for formants 1 and 2 over the interval of the stressed vowel using 4,500 Hz for male speakers and 5,500 Hz for female speakers as a ceiling. Since the formants vary substantially depending on the length of the vocal tract, we centered the formants for each speaker by subtracting the speaker mean.

To assess changes in voice quality, we measured the sound pressure level difference between the first and second harmonic H1^*^-H2^*^ (in dB) using a Long-Term Average Spectrum (LTAS) of the stressed vowel of the target word. We corrected both individual levels of H1 and H2 to control for the influence of the first two formants using the method introduced by Iseli et al. (2007) before subtraction. To express that the measure is a corrected version, we follow the convention to denote the quantity as H1^*^-H2^*^ with asterisks. Similarly, we calculated the sound pressure difference between the first harmonic and the highest sound pressure level in the vicinity of the third formant, H1^*^-A3^*^ (in dB), as a phonetic variable relating to the spectral balance of the stressed vowel. To determine A3, we extracted the mean formant 3 frequency in the stressed vowel and retrieved the maximum sound pressure level in a spectral window spanning ±10% of the formant frequency. Again, we used the method provided by Iseli et al. (2007), this time compensating for the influence of the first three formants. In addition, we measured HNR (harmonics-to-noise ratio) for the stressed vowel in the frequency range of 0 to 500 Hz using Praat's Harmonicity object.

Based on the intonation labels described in the previous section, the tonal onglide was measured as the distance in semitones between the beginning and the end of the accentual movement. The sign of the tonal onglide is indicative of the type or direction of accentual movement: Negative values are obtained for falling accents, positive values for rising accents. The absolute value reflects the magnitude of this movement.

In addition, we measured peak alignment as the time lag in ms between the stressed vowel onset and the maximum F0 point of the accent. In the case of rising accents, this maximum is the end of the movement; in the case of falling accents, this maximum is the beginning of the fall. In terms of an AM analysis, this maximum corresponds to an H tone (either H+ leading tone or H^*^).

As a further F0 variable, we measured target height, the distance in semitones between the end of the marked tonal movement, and the low boundary tone. This endpoint of the annotated movement corresponds to the starred tone in an AM analysis (e.g., H^*^ from L+H^*^ or !H^*^ from H+!H^*^). Since for some utterances no reliable F0 point at the low boundary could be found, we first calculated the mean F0 of all boundary tones of each speaker and used this means as a reference in the calculation.

The tonal onglide, peak alignment, and target height measures could only be obtained for accented target words since no movement can be described when the accent is absent. A measure that could be included for all four focus conditions in F0 mean, which is simply the mean F0 during the stressed vowel of the target word. To compensate for differences between male and female speakers, we measured F0 mean in semitones relative to the low boundary tone. As for the target height, we first calculated the average of all low boundary tones of each speaker and used this value as a reference. For all F0 calculations, we used the default To Pitch function of Praat with a range of 75 to 700 Hz.

From the signal of the backmost EMA sensor placed on the tongue, we extracted the average horizontal and vertical tongue position within the window of the first 50% of the acoustic vowel. Note that in the case of the horizontal tongue position, lower values indicate retracted tongue positions; in the case of the vertical tongue position, lower values indicate lowered tongue positions. In addition, we used the tangential velocity of the sensor to assess temporal aspects. Within the acoustic window of the article “die” preceding the stressed syllable of the target word, we determined the minimum of the tangential velocity as the start of the tongue movement (Jannedy et al., 2010). Within the acoustic window of the stressed vowel, we determined the minimum of the tangential velocity as the end of the tongue movement. We then calculated the tongue gesture duration based on these points (in ms).^5^ The maximum tangential velocity between the two points was taken as the tongue peak velocity (in mm/s). The interval between the start of the tongue movement and the peak velocity was calculated as the tongue time to peak velocity (in ms).

Moreover, we calculated the Euclidean distance between the upper and the lower lip sensors and determined the average Euclidean distance as the lip aperture (in mm) within the window of the first 50% of the acoustic vowel (analogous to the calculation of the tongue variables). All articulatory measures were performed with the emuR package. Since there is substantial physiological variation between speakers, we centered the spatial articulatory parameters (vertical and horizontal tongue position, and lip aperture) for each speaker by subtracting the speaker mean.

Finally, we used the ProPer suite (Albert et al., 2021) to extract the variables' periodic energy mass and synchrony. ProPer extracts smoothed periodic energy curves from the signal and quantify the area under the curve as the periodic energy mass in the interval corresponding to the stressed syllable. The variable synchrony represents the distance between the center of periodic energy-mass (CoM) and the center of gravity of the corresponding F0 trajectory (CoG). CoM and CoG follow a similar methodology in calculating a weighted average time point (based on the Tonal Center of Gravity, see Barnes et al., 2012, 2021), as shown in the Equations of (10) and (11).

(10) $CoM=\;\frac{\underset{i}{\sum }per_{i}t_{i}}{\underset{i}{\sum }per_{i}}$
(11) $CoG=\;\frac{\underset{i}{\sum }{F0}_{i}{per_{i}t}_{i}}{\underset{i}{\sum }{F0}_{i}per_{i}}$

Since the CoG reflects the F0 contour shape (fall vs. rise and convex vs. concave), the distance between CoG and CoM can provide information about the synchrony between the syllabic nuclei and the intonation contour. For instance, a rising tonal movement in the vowel is characterized by CoG following the CoM, while a falling movement is characterized by CoG preceding the CoM. Hence, while synchrony is indicative of the direction of the tonal movement, it also reflects its steepness or convexity. In the case of a rise, synchrony is expected to increase for a steeper or more convex movement (Cangemi et al., 2019). In this study, we report synchrony relative to the duration of the accented syllable in %.

### Statistical Modeling

To assess the role of the phonetic variables in marking focus structure we used Bayesian linear mixed models (for tutorial introductions with phonetic data, see Vasishth et al., 2018; Franke and Roettger, 2019; Nalborczyk et al., 2019). The models were fit with brms 2.16.3 (Bürkner, 2018) in R 4.1.2 (R Core Team, 2021). The package brms (‘Bayesian regression modeling with Stan') implements an interface to Stan, which is used to compute Bayesian models via Markov Chain Monte Carlo sampling (MCMC) (Carpenter et al., 2017). Throughout the analysis, we used tidyverse 1.3.1 for data processing (Wickham et al., 2019). For plotting, we used ggplot2 3.3.5 (Wickham, 2016) as included in tidyverse along with gridExtra 2.3 (Auguie, 2017) for the arrangement of plots.

We created subsets along two dimensions, the first one being focus type. To investigate the opposition of unaccented vs. accented, we compared target words in the background (unaccented) to target words in broad focus (accented). For this specific analysis, we did not include the variables onglide, alignment, target height, and synchrony since they relate to properties of pitch accents and there is no pitch accent on the target word in the background. A second subset was created to assess the differentiation of the accented focus types that contained only target words in broad, narrow, or contrastive focus.

Another subset dimension is vowel quality. We performed separate analyses for subsets of /a/ and /o/ as measures regarding formants and tongue position are very dependent on vowel quality, and different effects of prominence are expected to depend on the vowel (e.g., under accentuation the back vowel /o/ may be retracted while the central vowel /a/ may stay constant). All analyses are conducted for both vowels separately. Some recordings were missing due to technical problems or mispronunciations (46 productions); for some of the remaining recordings certain measures could not be obtained (4 productions; e.g., when a reliable F0 track in the region of interest was missing). We excluded all rows that contained missing values so that the same data were used in all models in each subset analysis. A total of 2110 data points entered the analysis (between 69 and 80 per speaker; 78 on average per speaker). To summarize, we have the following four subset analyses with the following numbers of observations:

1. accentuation /a/: target words with stressed /a/ in the background or in broad focus, *N* = 528
2. accentuation /o/: target words with stressed /o/ in the background or in broad focus, *N* = 520
3. focus types /a/: target words with stressed /a/ in broad focus, narrow focus or contrastive focus, *N* = 795
4. focus types /o/: target words with stressed /o/ in broad focus, narrow focus or contrastive focus, *N* = 787

All variables were z-scored (“standardized”) within each subset (i.e., subtracting the mean and dividing by the standard deviation separately for each variable). This way, all variables are more comparable: As a result of z-scoring, all variables have a mean of zero and a standard variation of 1. The variables lose their original unit (e.g., Hz) and are instead expressed in standard deviations.

We fitted separate Bayesian linear mixed models for each standardized variable. The model specifications were as follows: Each model contained focus type as the independent variable (predictor) and the phonetic measurement under consideration as the dependent variable (response). The models included random intercepts for speakers and target words, as well as random slopes for the effect of focus type by the speaker and by the target word. Four MCMC chains were run for 12,000 iterations with 5,000 warmup iterations, resulting in a total of 28,000 posterior samples used for inference.

We used a normally distributed prior with a mean of zero and an *SD* of 0.5 for all regression coefficients. All other priors were default priors of brms: For the intercept, a Student's *t* distribution was used with degrees of freedom of 3, a median of the data as mean of the distribution, and a standard deviation of 2.5 (ν = 3, μ = median of the data, σ = 2.5). As priors of the standard deviations of the random intercepts and slopes as well as the residual standard deviation of the model we used a Student's *t* distribution (ν = 3, μ = 0, σ = 2.5). The priors of the Cholesky factors of the covariance matrix for random effects were Cholesky LKJ correlation distributions (η = 1). The models were checked for convergence by ensuring no model yielded *Rhat* values larger than 1; model fits were assessed by visual inspection of the predictive posterior checks.^6^

In the following, we consider comparisons of the form “focus condition X is larger than focus condition Y” for each phonetic variable. For instance, we ask our model whether vowel duration is longer in contrastive focus than in broad focus. For such a comparison, we extract the following parameters from the model:

– β: The estimate of the mean difference between the focus types under consideration: mean of focus condition X minus mean of focus condition Y (e.g., contrastive focus minus broad focus)
– *SE*: The standard error of this estimate
– *LCI* and *HCI*: The high and low boundaries of the 90% credible interval (*CI*) of the estimate
– *Pr(*β >*0):* The probability that the estimate is above zero

This last quantity, the probability that the estimate is greater than zero, can also be formulated as the probability that the comparison “focus condition X > focus condition Y” is true. Note that some quantities decrease from focus condition X to focus condition Y. In these cases, a *Pr(*β >*0)* close to zero indicates that the probability of the opposite, namely “focus condition X < focus condition Y,” is high.

Since the variables are all standardized, the estimates of the mean differences also give an indication of the effect sizes of the different phonetic variables that are comparable across variables. To further assess the effect size, we provide Cohen's *d* as a statistic of effect size for each pair of focus types (e.g., broad vs. contrastive focus) calculated with the package effsize 0.8.1 (Torchiano, 2020). Prior to calculating Cohen's *d*, the values of each speaker were averaged. To distinguish it from the original Cohen's *d* calculation, we follow Westfall (2016) and denote it *d*_*a*_.

Finally, we calculated marginal and conditional *R*^2^ according to Nakagawa et al. (2017) using the r2_bayes() function of the performance package version 0.8.0 (Lüdecke et al., 2021). While conditional *R*^2^ provides information about the variance described by the fixed and random effects, marginal *R*^2^ indicates the variance described by the fixed effect alone. We report the *R*^2^ values in % for ease of interpretation.

### Data Availability

The data tables and analysis scripts of this study are publicly available and can be accessed here: https://osf.io/92ay8/.

## Results

### Descriptive Statistics

**Figure 1:**
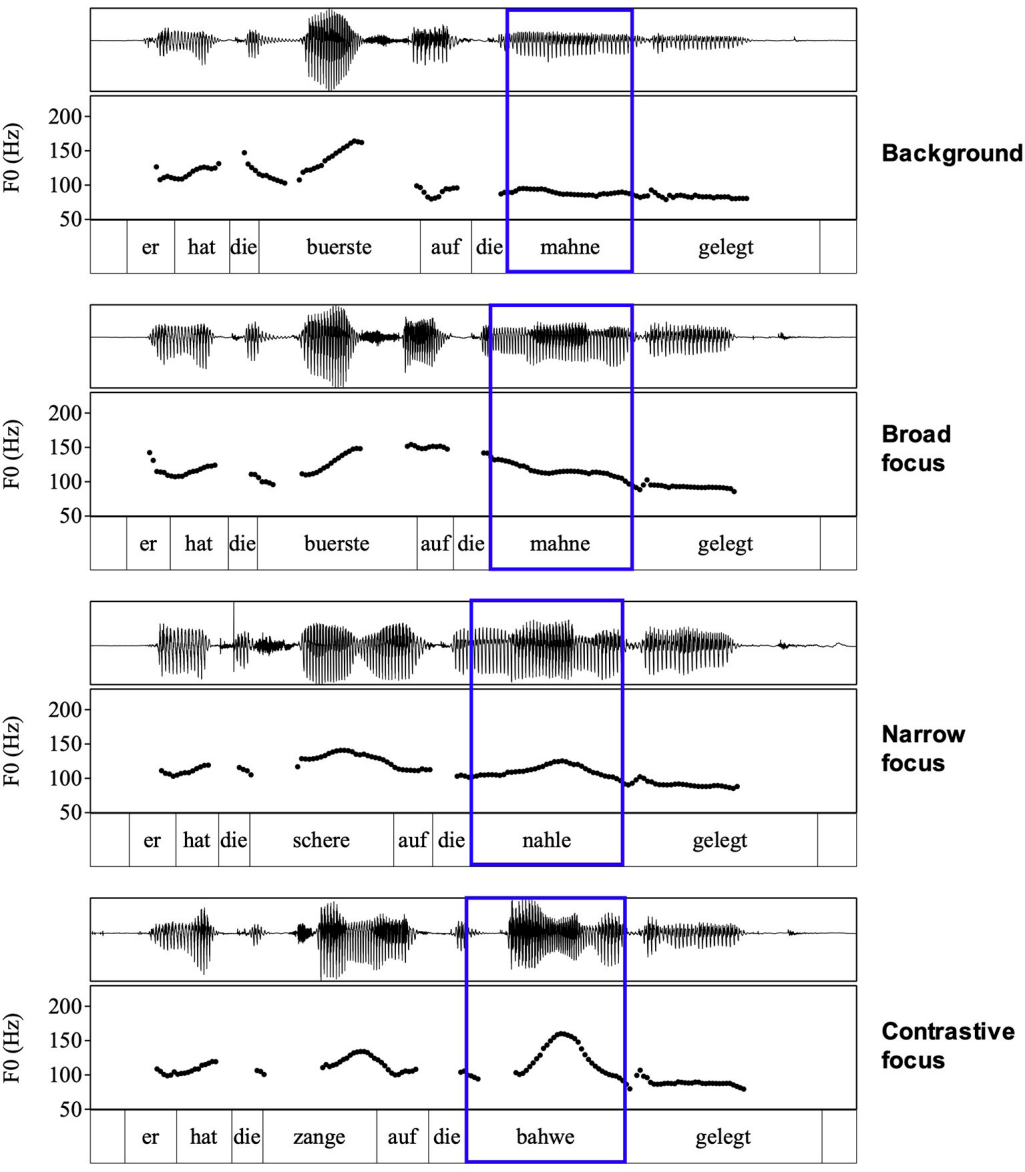
Examples for all four conditions.

Before turning to the modeling results, we provide waveform and F0 contours of four examples from one male speaker in Figure 1 for a more intuitive understanding of the data we are analyzing in this article. In each panel of the figure, the target word is marked by the blue box. In the background condition, when it is unaccented, it is characterized by a flat stretch of low F0 and rather low amplitude. Larger movements in the F0 track and higher amplitudes in the waveform can be observed for broad focus, narrow focus, and contrastive focus. In the case of broad focus, the F0 falls toward the center of the accented syllable while it rises in narrow focus and contrastive focus. In contrastive focus, the rising movement of the pitch accent is steeper and has a higher F0 target than in narrow focus (note that falling and rising accents are present in all three focus types but to different extents and that the rising movements show systematic variation in terms of steepness, see Roessig, 2021). These visualizations also illustrate why we did not include F0 measures to compare background to broad focus: F0 is low and flat in the target syllable while it is moving in broad focus. Measures like tonal onglide, peak alignment, and target height are not applicable in a strict sense to background items because these measures require F0 movement and the target words in the background condition have no pitch accent at all.

Table 1 provides the descriptive means of all variables for all focus conditions in non-standardized form (i.e., in the original unit). The four parameters which only apply to pitch accented syllables are given as N/A for the background condition. For almost all parameters, we see an increase from background to broad focus and from broad to contrastive focus; or a decrease from background to broad focus and from broad to contrastive focus. Exceptions are formant 2, horizontal tongue position in the vowel /a/, and HNR in both vowels. In many cases, the values for narrow focus are in between those for broad and contrastive focus.

**Table 1:** Descriptive means of all variables.

	**Vowel /a/**				**Vowel /o/**			
	**Background**	**Broad focus**	**Narrow focus**	**Contr. focus**	**Background**	**Broad focus**	**Narrow focus**	**Contr. focus**
F0 mean (semitones)	0.14	2.47	2.90	3.15	0.79	3.90	4.38	4.56
Tonal onglide (semitones)	N/A	−0.79	0.99	2.41	N/A	0.44	1.82	3.30
Peak alignment (ms)	N/A	6.84	82.98	124.73	N/A	14.04	70.79	103.13
Target height (semitones)	N/A	2.85	3.75	4.37	N/A	4.39	5.20	5.78
Synchrony (%)	N/A	−1.59	−0.51	0.72	N/A	−0.72	0.45	1.56
Vowel duration (ms)	154.46	170.02	174.75	176.88	123.84	136.13	141.31	140.74
RMS amplitude (mPa)	85.47	120.70	119.73	122.67	95.00	142.94	146.49	147.60
Periodic energy mass	124.59	187.10	199.53	196.53	111.79	169.19	183.26	178.38
H1*-A3* (dB)	16.31	15.83	15.85	14.71	10.25	7.89	7.61	6.42
H1*-H2* (dB)	6.51	8.59	9.83	10.76	6.77	7.68	8.29	8.59
HNR	26.83	28.76	28.86	28.52	26.84	28.62	28.72	27.95
Formant 1 (Hz, centered)	−53.91	6.84	20.75	26.91	−14.35	4.71	4.12	5.84
Formant 2 (Hz, centered)	−2.54	−3.20	−0.10	4.37	38.39	−2.68	−17.95	−22.03
Lip aperture (mm, centered)	2.06	3.44	3.51	4.06	−3.60	−3.31	−3.16	−3.02
Vertical tongue position (mm, centered)	−2.22	−2.76	−3.06	−3.26	3.29	2.86	2.70	2.45
Horizontal tongue position (mm, centered)	0.98	1.14	1.11	0.94	−0.62	−0.98	−1.22	−1.35
Tongue peak velocity (mm/s)	150.88	152.99	155.84	159.44	128.26	134.81	136.40	140.93
Tongue time to peak velocity (ms)	107.03	111.89	112.28	112.12	110.16	114.79	117.11	118.01
Tongue gesture duration (ms)	216.38	227.32	224.33	227.38	210.56	223.32	231.16	230.54

### Unaccented vs. Accented

This section presents the results of modeling the distinction between accented and unaccented syllables. They are based on the comparison “broad focus > background,” i.e., we ask: “Does each phonetic variable differ between background and broad focus?” The factor focus type has only two levels in this subset of the data (background and broad focus). We deliberately choose a broad focus for this comparison since it represents the “smallest” level of accentuation in our data. In contrast, many previous studies that investigated acoustic and articulatory parameters of prominence marking employed larger differences between focus structures, e.g., background vs. contrastive focus.

**Figure 2:**
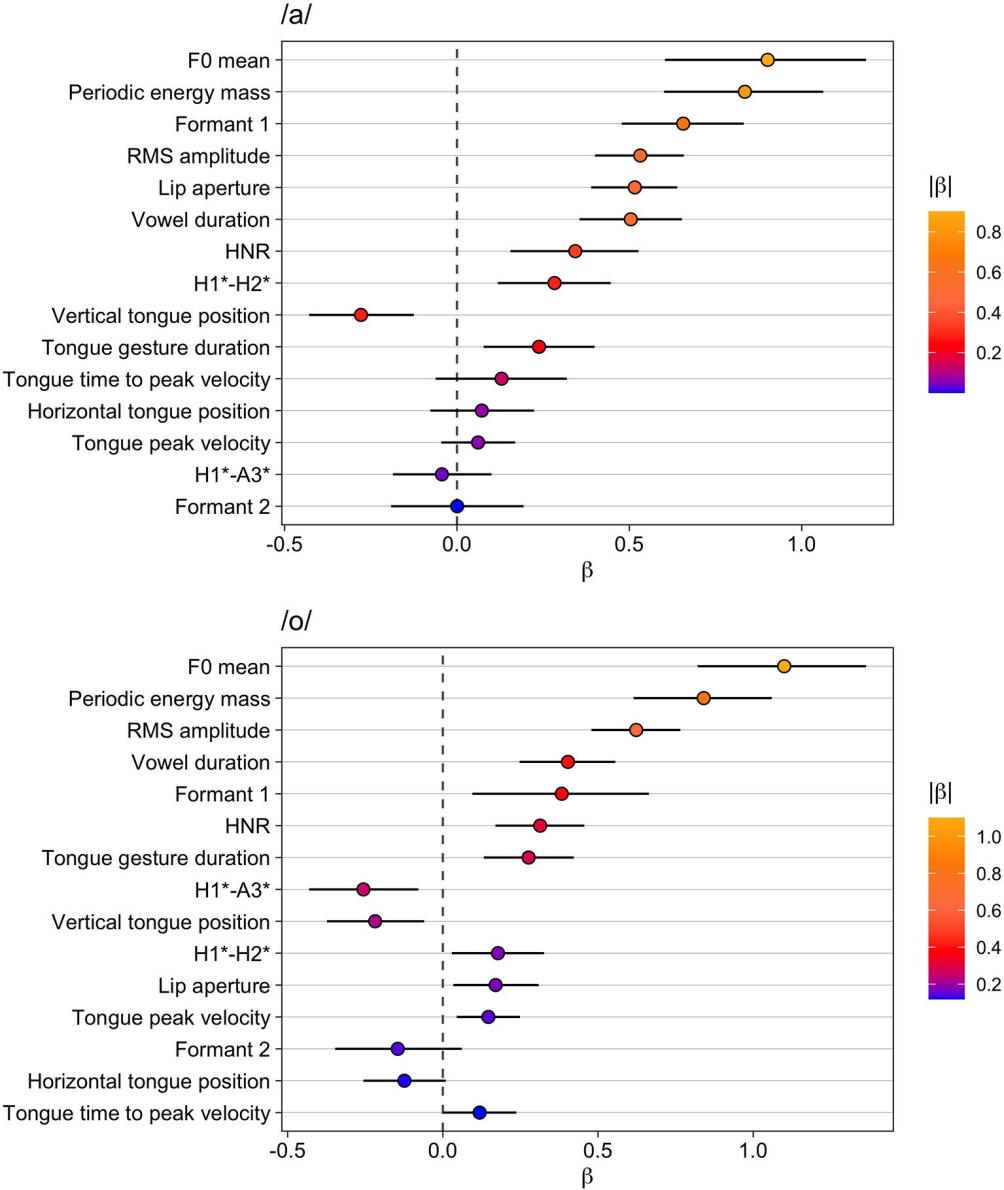
Modeling results for unaccented vs. accented. Top: vowel /a/, bottom: vowel /o/. The whiskers indicate the 90% Credible Interval.

The results for the vowel /a/ are given in Table 2 and the top panel of Figure 2. In both the table and the figure, the phonetic variables are arranged in descending order according to the absolute value of β. F0 mean increases in accented syllables and exhibits the largest difference between unaccented and accented syllables and the largest marginal *R*^2^ (fixed effects only). This phonetic parameter is closely followed by increased periodic energy-mass (which has an even larger effect size than F0 mean in terms of Cohen's *d*_a_). The increased periodic energy mass is related to RMS amplitude and lip aperture, two variables that also assume high ranks in the list (note that lip aperture shows a Cohen's *d*_a_ close to periodic energy mass despite being ranked below RMS amplitude). All these measures reflect higher intensities and the enhanced sonority of the accented vowel compared to the unaccented one.

**Table 2:** Tabular overview of modeling results for unaccented vs. accented, vowel /a/. LCI and HCI refer to the low and high boundaries of the 90% Credible Interval.

	**β**	**SE**	**LCI**	**HCI**	**Pr(β>0)**	***Cond. R* ^2^**	***Marg. R* ^2^**	**d_**a**_**
F0 mean	0.90	0.18	0.60	1.19	1.00	68.94	21.67	1.50
Periodic energy mass	0.84	0.14	0.60	1.06	1.00	65.15	18.10	1.69
Formant 1	0.66	0.11	0.48	0.83	1.00	19.86	10.93	2.00
RMS amplitude	0.53	0.08	0.40	0.66	1.00	92.16	7.16	0.59
Lip aperture	0.52	0.08	0.39	0.64	1.00	56.43	6.73	1.36
Vowel duration	0.50	0.09	0.36	0.65	1.00	71.55	6.38	0.66
HNR	0.34	0.11	0.15	0.53	1.00	50.93	2.96	0.51
Vertical tongue position	−0.28	0.09	−0.43	−0.13	0.00	31.82	1.95	0.53
H1*-H2*	0.28	0.10	0.12	0.45	1.00	32.08	2.01	0.49
Tongue gesture duration	0.24	0.10	0.08	0.40	0.99	45.66	1.43	0.37
Tongue time to peak velocity	0.13	0.12	−0.06	0.32	0.88	58.23	0.45	0.34
Horizontal tongue position	0.07	0.09	−0.08	0.22	0.79	37.73	0.17	0.14
Tongue peak velocity	0.06	0.07	−0.05	0.17	0.83	73.05	0.11	0.08
H1*-A3*	−0.04	0.09	−0.19	0.10	0.30	35.91	0.11	0.09
Formant 2	0.00	0.12	−0.19	0.19	0.50	41.76	0.15	0.01

Formant 1 is also ranked high (even higher than RMS amplitude) and is characterized by an increase in accented /a/—an outcome that is congruent with the result for the vertical tongue position and tongue gesture duration. For both of these variables, marginal *R*^2^ and *d*_a_ are much smaller than for Formant 1. Despite being smaller, their effects are robust as reflected by the posterior probabilities *Pr(*β >*0)*. Note that for vertical tongue position *Pr(*β >*0)* is 0 because the values of the variable decrease when the tongue is lowered. From this follows that the tongue position is consistently lower and the movement is longer in accented vowels as opposed to unaccented vowels. Vowel duration is ranked directly after RMS amplitude, indicating that vowels are longer and louder in accented syllables.

The voice quality variables HNR and H1^*^-H2^*^ show rather small effect sizes and low marginal *R*^2^ values (similar to the vertical tongue position), but both variables are consistently increased in accented syllables compared to unaccented syllables. This result could indicate a change from creaky voice to modal voice [remember that the literature revealed contrasting results in different studies as to whether H1^*^-H2^*^ increases or decreases under prominence, but the data here fit the model outlined in Garellek 2019 for a change from creaky to modal voice]. The results show no consistent impact on horizontal tongue position and formant 2 or on other temporal kinematic measures, as well as no influence on the spectral slope (H1^*^-A3^*^).

Table 3 and the bottom panel of Figure 2 present the results for the vowel /o/. Again, F0 mean has the largest difference between unaccented and accented syllables and the largest marginal *R*^2^. Periodic energy-mass (larger values for accented) follows and exhibits an even larger *d*_a_. RMS amplitude comes next with increased amplitudes in accented vowels. In addition, the model shows that vowels are longer when accented.

**Table 3:** Tabular overview of modeling results for unaccented vs. accented, vowel /o/. LCI and HCI refer to the low and high boundaries of the 90% Credible Interval.

	**β**	**SE**	**LCI**	**HCI**	**Pr(β>0)**	***Cond. R* ^2^**	***Marg. R* ^2^**	**d_**a**_**
F0 mean	1.10	0.16	0.82	1.36	1.00	86.44	32.91	1.74
Periodic energy mass	0.84	0.14	0.61	1.06	1.00	68.08	18.34	1.77
RMS amplitude	0.62	0.09	0.48	0.77	1.00	91.04	9.91	0.70
Vowel duration	0.40	0.09	0.25	0.56	1.00	69.61	4.11	0.57
Formant 1	0.38	0.17	0.09	0.66	0.98	24.66	3.73	0.85
HNR	0.31	0.09	0.17	0.46	1.00	57.19	2.48	0.42
Tongue gesture duration	0.28	0.09	0.13	0.42	1.00	56.37	1.91	0.37
H1*-A3*	−0.26	0.11	−0.43	−0.08	0.01	30.85	1.66	0.55
Vertical tongue position	−0.22	0.10	−0.37	−0.06	0.01	26.48	1.21	0.40
H1*-H2*	0.18	0.09	0.03	0.33	0.97	30.13	0.79	0.31
Lip aperture	0.17	0.08	0.03	0.31	0.98	34.60	0.72	0.31
Tongue peak velocity	0.15	0.06	0.04	0.25	0.99	69.15	0.54	0.19
Formant 2	−0.15	0.13	−0.35	0.06	0.12	15.35	0.57	0.52
Tongue time to peak velocity	0.12	0.07	0.00	0.24	0.95	52.73	0.35	0.22
Horizontal tongue position	−0.12	0.08	−0.26	0.01	0.06	40.05	0.39	0.31

Variables related to vowel articulation—formant 1, tongue gesture duration, vertical tongue position—are also affected consistently by accentuation in /o/: Formant 1 is higher and the tongue movement is longer and reaches a lower target. There is even a small effect on tongue peak velocity indicating that the movements also become slightly faster under accentuation. Furthermore, the estimate for the variable lip aperture shows a small but consistent positive effect, i.e., larger lip openings in accented /o/. The results for formant 2 and the horizontal tongue position are less clear. For horizontal tongue position, *Pr(*β >*0)* is 0.06, which means that given this data, our model specification, and our prior choice, the probability is indicated to be only 0.94 for the difference being negative, i.e., the tongue is more retracted. Thus, this difference is not particularly reliable, especially when compared to the others. The spectral slope variable H1^*^-A3^*^ decreases, but the effect does not seem to be very strong. The effect on the voice quality variable H1^*^-H2^*^ is even smaller and the evidence weaker with *Pr(*β >*0)* = 0.97 but taken together with the results for HNR that are stronger with larger effect size and a higher *Pr(*β>*0)* of 1, this outcome suggests less creaky, more modal voice in accented vowels compared to unaccented vowels.

Overall, the results for both vowels show the importance of F0 and of measures related to intensity (periodic energy mass, RMS amplitude). In addition, formant 1 and kinematic variables, such as lip aperture (at least for /a/) and vertical tongue position, are affected, but the effect sizes are smaller for these variables. Accentuation leads to longer vowel durations for both vowels, but with medium effect size. Finally, small effects can be attested for voice quality and spectral slope (only for /o/). Overall, the results are comparable for both /a/ and /o/.

### Focus Types: Broad, Narrow, and Contrastive

In this section, we investigate how the focus types broad, narrow and contrastive are distinguished. They are all characterized by the fact that the nuclear accent is placed on the target word. This means that all measures that are compared here are from nuclear-accented syllables. The factor focus type has three distinct levels in the models of this sub-analysis (broad focus, narrow focus, and contrastive focus) so that we have three comparisons (broad vs. contrastive; broad vs. narrow; narrow vs. contrastive). Since the *R*^2^ quantities apply to each model as a whole, we present them separately in Table 4 ordered by marginal *R*^2^ (fixed effects only) descending order from top to bottom. This table contains the values for both vowels.

**Table 4:** Conditional and marginal *R*^2^ of the focus types models (expressed in %).

**Vowel /a/**			**Vowel /o/**		
	***Cond. R* ^2^**	***Marg. R* ^2^**		***Cond. R* ^2^**	***Marg. R* ^2^**
Peak alignment	43.68	11.50	Tonal onglide	63.07	11.38
Tonal onglide	54.63	10.41	Peak alignment	48.37	9.38
Synchrony	48.04	6.73	Synchrony	49.62	8.53
Target height	71.91	3.40	Target height	83.01	2.81
H1*-H2*	36.99	1.94	Vertical tongue position	32.86	1.01
Vertical tongue position	30.50	1.36	F0 mean	83.71	0.95
Formant 1	8.85	1.19	Periodic energy mass	63.87	0.90
Lip aperture	57.06	1.13	Tongue gesture duration	59.58	0.69
F0 mean	72.78	1.06	H1*-H2*	40.13	0.65
Vowel duration	71.59	0.83	Vowel duration	70.89	0.62
Periodic energy mass	60.50	0.77	H1*-A3*	34.09	0.62
H1*-A3*	44.98	0.41	Horizontal tongue position	39.65	0.48
Tongue peak velocity	73.35	0.33	HNR	61.61	0.43
Horizontal tongue position	39.42	0.28	Lip aperture	36.49	0.39
HNR	51.74	0.22	Formant 2	12.90	0.29
Tongue gesture duration	49.08	0.19	Tongue peak velocity	67.49	0.26
Formant 2	40.94	0.18	Formant 1	4.09	0.25
Tongue time to peak velocity	59.32	0.12	Tongue time to peak velocity	58.33	0.19
RMS amplitude	91.09	0.06	RMS amplitude	88.62	0.17

The models with tonal onglide and peak alignment yield the highest marginal *R*^2^, followed by synchrony and target height. This underlines the importance of F0 for the encoding of focus structure. Interestingly, the other variable related to F0, which is the F0 mean, is found in the mid regions of both lists, indicating that this measure fails to capture a substantial part of the contribution of F0. Some kinematic parameters yield medium values of marginal *R*^2^: Vertical tongue position for /o/; and lip aperture and vertical tongue position for /a/. These results for /a/ correspond to the result obtained for formant 1 which directly follows in the list. Another phonetic variable that is located in the upper part of the list is the voice quality parameter H1^*^-H2^*^ that directly follows the F0 variables for the vowel /a/. However, HNR is ranked substantially lower weakening the role of voice quality for the differentiation of focus types. Interestingly, RMS amplitude, which yielded rather high marginal *R*^2^ for the modeling of unaccented vs. accented, ranks low for both vowels in the present models.

#### Broad vs. Contrastive Focus

**Figure 3:**
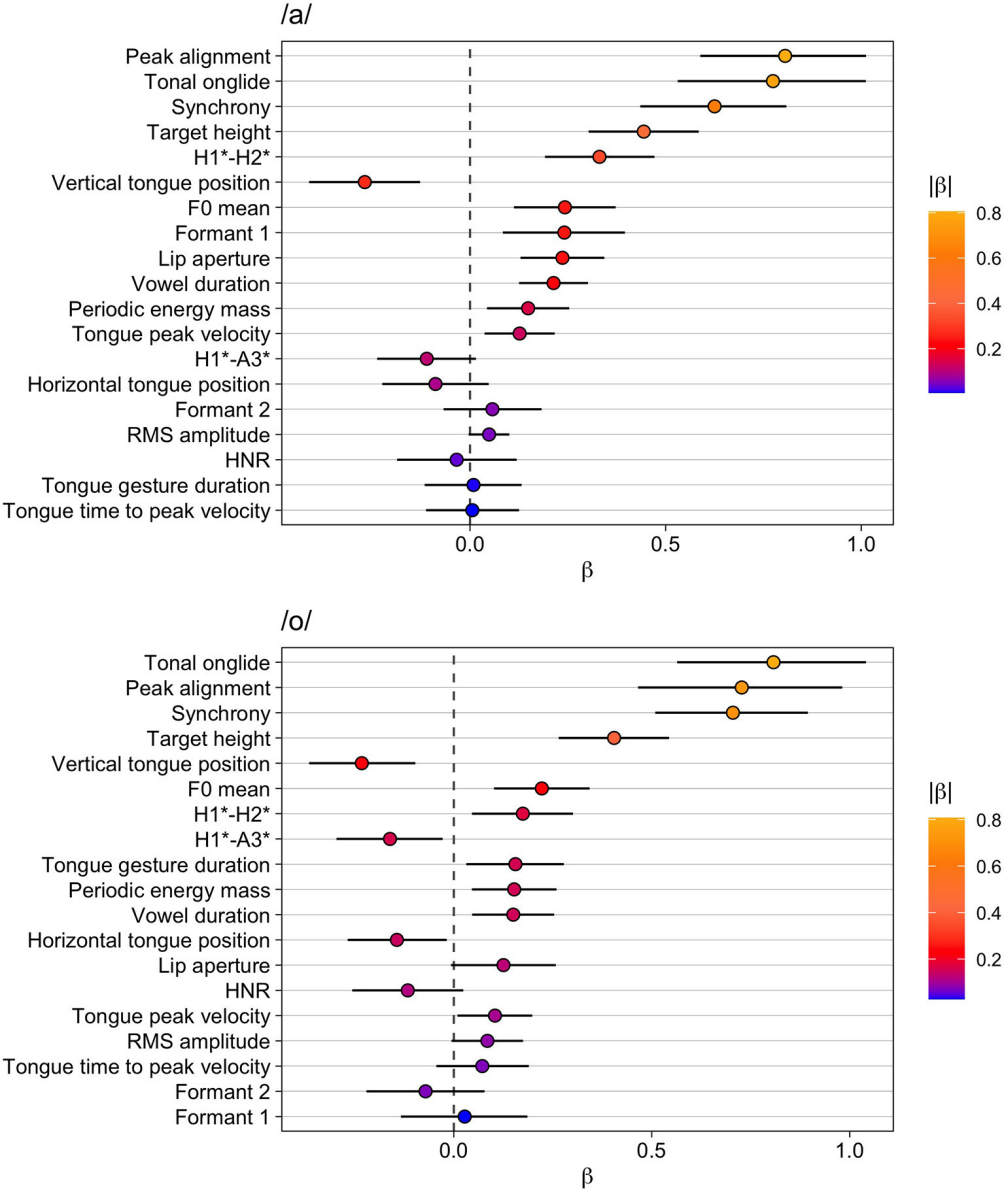
Modeling results for broad vs. contrastive focus. Top: vowel /a/, bottom: vowel /o/. The whiskers indicate the 90% Credible Interval.

For the vowel /a/, the results are presented in Table 5 and in the upper panel of Figure 3. The models reveal the strongest effects for peak alignment, tonal onglide, synchrony, and target height—all of which are phonetic parameters that describe the F0 movement of the nuclear pitch accent. Later alignments, higher tonal onglides, later synchrony, and larger target heights are found in contrastive focus compared to broad focus. The additional variable characterizing F0, which is the F0 mean, occurs at a lower position in the list. With regard to voice quality, the results show consistently increased H1^*^-H2^*^ but HNR does not change. Furthermore, the articulatory variables vertical tongue position and lip aperture show consistent effects toward more displaced kinematic targets (tongue is lowered, lips are opened wider). This kinematic finding is reflected in higher formant 1 values. Vowel duration yields a robust result with *Pr(*β >*0)* of 1.00 indicating longer vowels in contrastive focus compared to broad focus, but the effect size is rather small. Similarly, periodic energy-mass (larger mass in contrastive focus) and tongue peak velocity (faster tongue movements in contrastive focus) show small effects.

**Table 5:** Tabular overview of modeling results for broad vs. contrastive focus, vowel /a/. LCI and HCI refer to the low and high boundaries of the 90% Credible Interval.

	**β**	**SE**	**LCI**	**HCI**	**Pr(β>0)**	**d_**a**_**
Peak alignment	0.81	0.13	0.59	1.01	1.00	1.41
Tonal onglide	0.77	0.15	0.53	1.01	1.00	1.28
Synchrony	0.63	0.11	0.44	0.81	1.00	1.07
Target height	0.44	0.09	0.30	0.58	1.00	0.56
H1*-H2*	0.33	0.09	0.19	0.47	1.00	0.55
Vertical tongue position	−0.27	0.09	−0.41	−0.13	0.00	0.47
F0 mean	0.24	0.08	0.11	0.37	1.00	0.30
Formant 1	0.24	0.10	0.08	0.40	0.99	0.87
Lip aperture	0.24	0.07	0.13	0.34	1.00	0.56
Vowel duration	0.21	0.05	0.13	0.30	1.00	0.26
Periodic energy mass	0.15	0.06	0.04	0.25	0.99	0.25
Tongue peak velocity	0.13	0.06	0.04	0.22	0.99	0.15
H1*-A3*	−0.11	0.08	−0.24	0.02	0.07	0.18
Horizontal tongue position	−0.09	0.08	−0.22	0.05	0.14	0.16
Formant 2	0.06	0.08	−0.07	0.18	0.77	0.23
RMS amplitude	0.05	0.03	0.00	0.10	0.94	0.05
HNR	−0.03	0.09	−0.19	0.12	0.35	0.06
Tongue gesture duration	0.01	0.08	−0.12	0.13	0.55	0.00
Tongue time to peak velocity	0.01	0.07	−0.11	0.13	0.53	0.01

The results for /o/ are given in Table 6 and the lower part of Figure 3. Again, tonal onglide, peak alignment, synchrony, and target height are at the top of the list and the F0 mean exhibits a smaller effect. This time, the effect on tonal onglide is slightly larger than on peak alignment, but the two variables are very close. All in all, the estimates for the F0 parameters show that contrastive focus is characterized by larger tonal onglides, later peak alignments and synchrony values, as well as higher F0 targets and F0 means.

**Table 6:** Tabular overview of modeling results for broad vs. contrastive focus, vowel /o/. LCI and HCI refer to the low and high boundaries of the 90% Credible Interval.

	**β**	**SE**	**LCI**	**HCI**	**Pr(β>0)**	**d_**a**_**
Tonal onglide	0.81	0.15	0.56	1.04	1.00	1.25
Peak alignment	0.73	0.16	0.47	0.98	1.00	1.26
Synchrony	0.71	0.12	0.51	0.89	1.00	1.18
Target height	0.40	0.08	0.26	0.54	1.00	0.49
Vertical tongue position	−0.23	0.08	−0.37	−0.10	0.00	0.44
F0 mean	0.22	0.07	0.10	0.34	1.00	0.27
H1*-H2*	0.17	0.08	0.05	0.30	0.99	0.28
Tongue gesture duration	0.16	0.08	0.03	0.28	0.98	0.21
H1*-A3*	−0.16	0.08	−0.30	−0.03	0.03	0.37
Vowel duration	0.15	0.06	0.05	0.25	0.99	0.22
Periodic energy mass	0.15	0.07	0.05	0.26	0.99	0.33
Horizontal tongue position	−0.14	0.08	−0.27	−0.02	0.03	0.33
Lip aperture	0.13	0.08	−0.01	0.26	0.94	0.27
HNR	−0.12	0.09	−0.26	0.02	0.09	0.13
Tongue peak velocity	0.10	0.06	0.01	0.20	0.96	0.12
RMS amplitude	0.08	0.06	−0.01	0.17	0.94	0.09
Tongue time to peak velocity	0.07	0.07	−0.04	0.19	0.85	0.19
Formant 2	−0.07	0.09	−0.22	0.08	0.21	0.29
Formant 1	0.03	0.10	−0.13	0.19	0.61	0.14

The vertical tongue position is ranked relatively high and follows directly after the variable target height, but the effect size is quite small. Other kinematic variables (tongue gesture duration, horizontal tongue position, tongue peak velocity) yield rather robust results (with *Pr(*β >*0)* > 0.95 in the case of positive estimated differences or *Pr(*β >*0)* < 0.05 in the case of negative estimated differences)—but effect sizes are rather small as is apparent from the low absolute values of β and *d*_*a*_. Perhaps surprisingly, the formant values do not seem to be affected systematically. H1^*^-A3^*^ decreases indicating a shallower spectral tilt for contrastive focus compared to broad focus, i.e., more energy in high frequencies in contrastive focus. (Note that *Pr(*β >*0)* is close to zero which means that there is a high probability that the difference between broad and contrastive focus is negative.) H1^*^-H2^*^ increases, but—similar to the results for /a/—no consistent effect for HNR can be attested leaving the results for voice quality inconclusive (in fact, the estimate of HNR is negative indicating more noise in the signal, but the *Pr(*β >*0)* of 0.09 does not provide strong evidence for a decrease of the variable). Finally, vowel duration and periodic energy mass show small but consistent positive effects with *Pr(*β>*0)* = 0.99 (longer durations and larger masses in contrastive focus compared to broad focus). For lip aperture and RMS amplitude, small positive effects are found but *Pr(*β >*0)* is only 0.94 in both cases.

#### Broad vs. Narrow Focus

**Figure 4:**
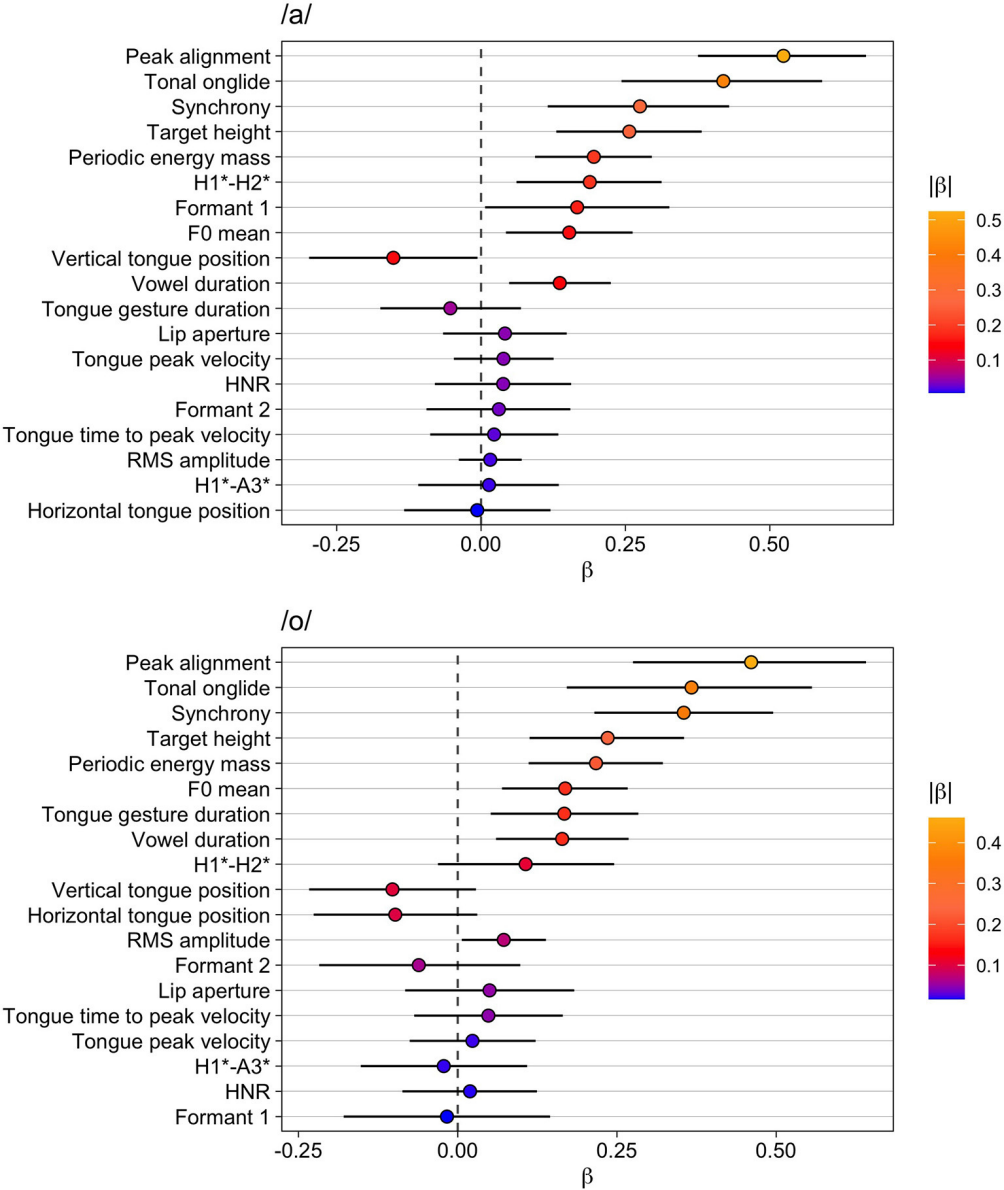
Modeling results for broad vs narrow focus. Top: vowel /a/, bottom: vowel /o/. The whiskers indicate the 90% Credible Interval.

Table 7 and the top panel of Figure 4 present the results for broad vs. narrow focus for the vowel /a/. Peak alignment is again at the top of the list, followed by tonal onglide, synchrony, and target height. Since all estimates are positive, we can conclude that narrow focus leads to later alignments, higher tonal onglides, later synchrony, and larger target heights. Periodic energy mass and H1^*^-H2^*^ are next in the list with increased values for narrow focus. The findings for H1^*^-H2^*^ may be indicative of a trend away from creaky voice and toward modal voice but this is not supported by the estimates for HNR that suggest that this variable is not affected systematically between broad and narrow focus.

**Table 7:** Tabular overview of modeling results for broad vs. narrow focus, vowel /a/. LCI and HCI refer to the low and high boundaries of the 90% Credible Interval.

	**β**	**SE**	**LCI**	**HCI**	**Pr(β>0)**	**d_**a**_**
Peak alignment	0.52	0.09	0.38	0.67	1.00	0.88
Tonal onglide	0.42	0.11	0.24	0.59	1.00	0.73
Synchrony	0.28	0.10	0.12	0.43	1.00	0.50
Target height	0.26	0.08	0.13	0.38	1.00	0.34
Periodic energy mass	0.20	0.06	0.09	0.30	1.00	0.31
H1*-H2*	0.19	0.08	0.06	0.31	0.99	0.32
Formant 1	0.17	0.10	0.01	0.33	0.96	0.60
Vertical tongue position	−0.15	0.09	−0.30	−0.01	0.04	0.30
F0 mean	0.15	0.07	0.04	0.26	0.99	0.19
Vowel duration	0.14	0.05	0.05	0.22	0.99	0.17
Tongue gesture duration	−0.05	0.08	−0.17	0.07	0.23	0.09
Lip aperture	0.04	0.06	−0.07	0.15	0.74	0.09
Tongue peak velocity	0.04	0.05	−0.05	0.13	0.78	0.06
HNR	0.04	0.07	−0.08	0.16	0.71	0.04
Formant 2	0.03	0.08	−0.09	0.15	0.66	0.10
Tongue time to peak velocity	0.02	0.07	−0.09	0.13	0.64	0.02
RMS amplitude	0.02	0.03	−0.04	0.07	0.69	0.02
Horizontal tongue position	−0.01	0.08	−0.13	0.12	0.47	0.02
H1*-A3*	0.01	0.07	−0.11	0.13	0.58	0.00

In addition, the list reveals higher formant 1 values and lower vertical tongue positions for /a/ in narrow focus. However, for both of these variables, *Pr(*β >*0)* is less extreme with 0.96 and 0.04, which indicates that these results are not as reliable as the ones obtained for the other phonetic parameters outlined above. F0 mean again assumes a middle rank in the list, with a positive effect that is weaker than the other F0 parameters, especially compared to peak alignment and tonal onglide. Finally, vowel duration shows a small but rather consistent effect with longer vowel durations in narrow focus.

Table 8 and the bottom panel of Figure 4 present the results for the vowel /o/. As seen before, the four F0 parameters peak alignment, tonal onglide, synchrony, and target height are at the top of the list with positive effects. F0 mean exhibits a smaller yet robust effect and is located in a mid-rank position on the list. Periodic energy mass, also in the middle of the list, is directly following target height. Tongue gesture duration is the only kinematic variable with a small but robust effect; position parameters and formants are not affected systematically. Finally, the result for vowel duration indicates slightly longer vowels in narrow compared to broad focus.

**Table 8:** Tabular overview of modeling results for broad vs. narrow focus, vowel /o/. LCI and HCI refer to the low and high boundaries of the 90% Credible Interval.

	**β**	**SE**	**LCI**	**HCI**	**Pr(β>0)**	**d_**a**_**
Peak alignment	0.46	0.11	0.28	0.64	1.00	0.72
Tonal onglide	0.37	0.12	0.17	0.56	1.00	0.69
Synchrony	0.35	0.09	0.21	0.50	1.00	0.61
Target height	0.24	0.07	0.11	0.36	1.00	0.30
Periodic energy mass	0.22	0.06	0.11	0.32	1.00	0.44
Tongue gesture duration	0.17	0.07	0.05	0.28	0.99	0.21
F0 mean	0.17	0.06	0.07	0.27	1.00	0.20
Vowel duration	0.16	0.06	0.06	0.27	0.99	0.23
H1*-H2*	0.11	0.09	−0.03	0.25	0.90	0.18
Vertical tongue position	−0.10	0.08	−0.23	0.03	0.10	0.20
Horizontal tongue position	−0.10	0.08	−0.23	0.03	0.10	0.20
RMS amplitude	0.07	0.04	0.01	0.14	0.96	0.08
Formant 2	−0.06	0.10	−0.22	0.10	0.26	0.18
Lip aperture	0.05	0.08	−0.08	0.18	0.74	0.15
Tongue time to peak velocity	0.05	0.07	−0.07	0.17	0.76	0.15
Tongue peak velocity	0.02	0.06	−0.08	0.12	0.65	0.03
Formant 1	−0.02	0.10	−0.18	0.14	0.43	0.01
H1*-A3*	−0.02	0.08	−0.15	0.11	0.39	0.06
HNR	0.02	0.06	−0.09	0.12	0.62	0.05

#### Narrow vs. Contrastive Focus

**Figure 5:**
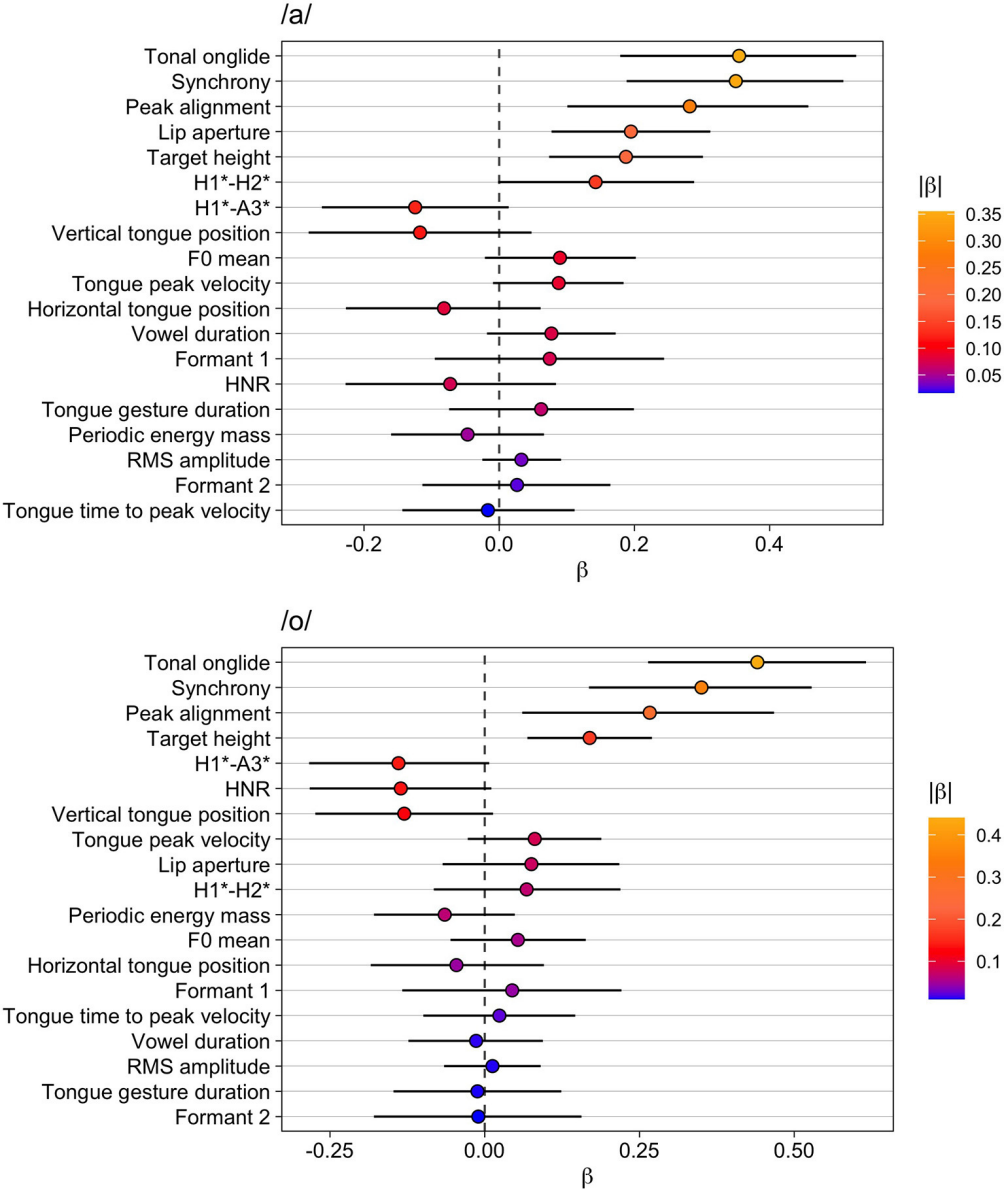
Modeling results for narrow vs. contrastive focus. Top: vowel /a/, bottom: vowel /o/. The whiskers indicate the 90% Credible Interval.

The results for /a/ are given in Table 9 and the upper part of Figure 5. Tonal onglide exhibits the biggest difference between narrow and contrastive focus and is closely followed by synchrony and peak alignment. Interestingly, the variable lip aperture ranks higher than target height indicating that the lips are opened wider in contrastive focus than in narrow focus, potentially leading to an increase in the vowel's sonority. H1^*^-H2^*^ changes in the same direction as it does from background to broad focus, broad to contrastive focus, and broad to narrow focus but the effect is small and P(β >0) is only 0.95. There is no evidence for a systematic modification of HNR. The spectral slope variable H1^*^-A3^*^ seems to be affected slightly. However, P(β >0) is 0.07, i.e., the probability that the parameter indeed decreases is only 0.93, indicating that this result is not particularly reliable.

**Table 9:** Tabular overview of modeling results for narrow versus contrastive focus, vowel /a/. LCI and HCI refer to the low and high boundaries of the 90% Credible Interval.

	**β**	**SE**	**LCI**	**HCI**	**Pr(β>0)**	**d_**a**_**
Tonal onglide	0.35	0.11	0.18	0.53	1.00	0.53
Synchrony	0.35	0.10	0.19	0.51	1.00	0.53
Peak alignment	0.28	0.11	0.10	0.46	0.99	0.57
Lip aperture	0.19	0.07	0.08	0.31	1.00	0.49
Target height	0.19	0.07	0.07	0.30	0.99	0.21
H1*-H2*	0.14	0.09	0.00	0.29	0.95	0.24
Vertical tongue position	−0.12	0.10	−0.28	0.05	0.12	0.21
H1*-A3*	−0.12	0.08	−0.26	0.01	0.07	0.18
Tongue peak velocity	0.09	0.06	−0.01	0.18	0.93	0.10
F0 mean	0.09	0.07	−0.02	0.20	0.91	0.10
Horizontal tongue position	−0.08	0.09	−0.23	0.06	0.17	0.14
Vowel duration	0.08	0.06	−0.02	0.17	0.91	0.09
Formant 1	0.07	0.10	−0.10	0.24	0.77	0.23
HNR	−0.07	0.10	−0.23	0.08	0.21	0.10
Tongue gesture duration	0.06	0.08	−0.07	0.20	0.78	0.09
Periodic energy mass	−0.05	0.07	−0.16	0.07	0.24	0.07
Formant 2	0.03	0.08	−0.11	0.16	0.63	0.17
RMS amplitude	0.03	0.04	−0.02	0.09	0.83	0.03
Tongue time to peak velocity	−0.02	0.08	−0.14	0.11	0.41	0.02

Table 10 and the lower panel of Figure 5 display the results for /o/. The biggest difference between narrow and contrastive focus is observed for tonal onglide. Apart from this effect, robust effects were only obtained for synchrony, peak alignment, and target height. Spectral slope (H1^*^-A3^*^) and HNR seem to be affected slightly, but *Pr(*β >*0)* is 0.06 in both cases, i.e., the probability that the parameters indeed decrease is only 0.94. Interestingly, HNR decreases indicating a larger proportion of inharmonic signal but, as pointed out, the effect is small and the model provides weak evidence for a systematic decrease of this variable. In addition, there is a small negative effect on the vertical tongue position, but *Pr(*β >*0)* = 0.07 indicates that this result is also not particularly reliable.

**Table 10:** Tabular overview of modeling results for narrow vs. contrastive focus, vowel /o/. LCI and HCI refer to the low and high boundaries of the 90% Credible Interval.

	**β**	**SE**	**LCI**	**HCI**	**Pr(β <0)**	**d_**a**_**
Tonal onglide	0.44	0.11	0.26	0.62	1.00	0.59
Synchrony	0.35	0.11	0.17	0.53	1.00	0.54
Peak alignment	0.27	0.12	0.06	0.47	0.98	0.58
Target height	0.17	0.06	0.07	0.27	1.00	0.18
H1*-A3*	−0.14	0.09	−0.28	0.01	0.06	0.28
HNR	−0.14	0.09	−0.28	0.01	0.06	0.18
Vertical tongue position	−0.13	0.09	−0.27	0.01	0.07	0.23
Tongue peak velocity	0.08	0.07	−0.03	0.19	0.89	0.09
Lip aperture	0.08	0.09	−0.07	0.22	0.81	0.12
H1*-H2*	0.07	0.09	−0.08	0.22	0.78	0.10
Periodic energy mass	−0.06	0.07	−0.18	0.05	0.17	0.11
Horizontal tongue position	−0.05	0.09	−0.18	0.10	0.29	0.11
F0 mean	0.05	0.07	−0.06	0.16	0.80	0.06
Formant 1	0.04	0.11	−0.13	0.22	0.67	0.16
Tongue time to peak velocity	0.02	0.08	−0.10	0.15	0.63	0.04
Tongue gesture duration	−0.01	0.08	−0.15	0.12	0.44	0.02
Vowel duration	−0.01	0.07	−0.12	0.09	0.41	0.01
Formant 2	−0.01	0.10	−0.18	0.16	0.46	0.05
RMS amplitude	0.01	0.05	−0.07	0.09	0.61	0.01

## Discussion

Our results demonstrate that focus is expressed by a multitude of phonetic parameters. This is true for the comparison of background vs. broad focus in which the placement of the nuclear pitch accent is changed, but also for the comparisons between the focus types broad, narrow and contrastive with the nuclear pitch accent in the same position. The modeling approach that we employed in this study makes it possible to compare effect sizes on the 19 different phonetic variables since they are standardized.

The results provide the highest number of reliable effects and largest effect sizes for background vs. broad focus and for broad vs. contrastive focus, while the differences between narrow focus and the other two focus types, broad and contrastive, are less consistent and more subtle. Many of the effects that are found for background vs. broad focus and for broad vs. contrastive focus go in the same direction in the comparisons involving narrow focus. This result indicates that narrow focus generally lies in between broad and contrastive focus for most phonetic variables. For some phonetic variables, narrow focus is similar to broad focus (e.g., lip aperture for /a/), for others it resembles contrastive focus (e.g., vowel duration for /o/). Yet, for other phonetic variables, all three types—broad, narrow, and contrastive focus—are differentiated (e.g., tonal onglide).

With respect to the relative importance of the different phonetic variables, the results show that F0 is most strongly affected and thereby confirm the importance of F0 for prosodic prominence (Cooper et al., 1985; Pierrehumbert and Hirschberg, 1990; Rump and Collier, 1996; Baumann et al., 2007; Breen et al., 2010). For background vs. broad focus, this is demonstrated by the fact that the F0 mean ranks highest in terms of the coefficient size. For broad, narrow, and contrastive focus, the importance of F0 is particularly evident because tonal onglide, peak alignment, synchrony, and target height all rank amongst the most influential phonetic variables.

The F0 results also show how important the method of quantification is. In the differentiation of the focus types broad, narrow and contrastive, the effect of the variable F0 mean is less strong compared to tonal onglide, peak alignment, synchrony, and target height. In contrast to F0 mean, the other four parameters take important aspects of the F0 contour into account (direction and magnitude of movement, temporal alignment to segments, contour shape). This demonstrates the importance of using more complex F0 measures rather than just F0 mean (cf. Grice et al., 2017).

Another interesting pair of parameters are RMS amplitude and periodic energy mass. The two measures are closely related since higher periodic energy should be correlated with higher RMS amplitudes in vowels. Interestingly, periodic energy mass ranks higher than RMS amplitude in all comparisons. A possible explanation is that the periodic energy mass, integrating the area under the periodic energy curve, incorporates the temporal domain. In doing so, it may be able to reflect the joint contribution of duration and intensity that was found to be important in the perception of prominence by Turk and Sawusch (1996). Interestingly, while both RMS amplitude and periodic energy mass are ranked high for unaccented vs. accented, their roles are only minor in the differentiation of broad, narrow, and contrastive focus (this is especially true for RMS amplitude). Thus, our results are in line with the literature showing that intensity is modulated under accentuation (Fowler, 1995; Harrington et al., 2000). Our study goes beyond these results in suggesting that intensity modulation is primarily a marker of accentuation, and plays a less important role for prominence gradations when an accent is present.

Vowel duration is consistently longer in accented compared to unaccented words. It is also a phonetic variable used to distinguish focus types, as it is found to be longer in contrastive focus compared to broad focus, and in narrow focus compared to broad focus. Thus, the narrow focus seems to pattern with a contrastive focus in terms of vowel duration. Although our results confirm the role of duration for prominence (Cooper et al., 1985; Turk and Sawusch, 1996; Breen et al., 2010; Mücke and Grice, 2014), it has to be noted that the effect sizes of vowel duration are rather small overall and the variable assumes a middle rank in the lists. As one reviewer pointed out, the rather small effect sizes of duration in our analysis may be explained by the fact that other phonetic variables, like F0, are permitted to vary to a large extent in West-Germanic intonation languages. In tone languages, in which F0 is already used to mark lexical differences and its use in the marking of focus types is hence more restricted, duration may be promoted to play a more important role (DiCanio et al., 2018).

Furthermore, the results of this study support previous findings showing that kinematic properties of the supra-laryngeal articulation are indeed affected by focus structure (Beckman et al., 1992; de Jong, 1995; Harrington et al., 2000; Cho, 2005; Mücke and Grice, 2014). While modifications are found in accentuation (background vs. broad) and in the differentiation of broad and contrastive focus, supra-laryngeal kinematics seem to play a more important role for the former than for the latter: within the group of accented focus types, the modifications are more subtle compared to the modifications under accentuation. A possible explanation may be that the articulator positions are already rather peripheral due to accentuation and there is only reduced space left for modifications in the accented group. Additional hyperarticulatory modifications in the same direction would potentially lead to less intelligible renditions of the vowels (which would be the opposite of the assumed speaker intention). This might be especially true for /o/ which has a neighboring vowel category positioned lower in the space while there is no lower neighbor of /a/.

With regard to the relative impact of focus on the different articulatory variables, the effects are strongest for the lip aperture during the vowel and the vertical tongue position. Prosodic prominence hence leads to larger lip openings and lower tongue positions. The effect for lip opening is much stronger in /a/ than in /o/ which could be due to the fact that the vowel /o/ involves lip rounding that counteracts the maximization of lip aperture. The results for formants and articulatory variables are not always congruent, which may point to the non-linearity between articulatory vocal tract configurations and the acoustic outcome of a complex filter system. Potentially, these results may also shed light on the debate as to whether articulation or acoustics exhibit more variability (Johnson et al., 1993; Whalen et al., 2018). It is not clear what counts as variability, since prosody-induced variation, as observed here in a controlled manner, may often be subsumed under general variability. If we view prosody-induced changes as mere variability, then our results point toward less variable acoustics. If we view prosody-induced changes as systematic, our results may point toward stable articulatory patterns whereas the acoustic effects are less consistent. Hence, our findings show that we need to sharpen our understanding of variability and think about its sources.

The results for the variable H1^*^-A3^*^ indicate that there is some effect of prosodic prominence on the spectral slope for the vowel /o/: the variable is consistently lower in broad focus compared to the background, and in contrastive compared to broad focus. For narrow vs. contrastive focus the results are weaker. Lowering of this variable indicates that the spectral slope becomes flatter, i.e., there is more energy around the third formant in the high-frequency bands of the spectrum compared to the F0. The finding of more energy in higher frequency bands is in line with the results provided in the literature on a spectral slope in the production and perception of prosodic prominence (Sluijter et al., 1995; Campbell and Beckman, 1997; Cole et al., 2010; Baumann and Winter, 2018).

We obtain consistently raised H1^*^-H2^*^ and HNR values for accented vs. unaccented vowels. As outlined in the section on the background of this study, the literature presents contradictory results as to whether this parameter increases or decreases under prominence (Campbell and Beckman, 1997; Epstein, 2003). In light of Ladefoged's (1980) model, both directions may be interpreted as a trend toward the modal voice in prominent vowels from either side of the continuum—breathy or creaky voice (Keating and Esposito, 2007). With the extension of this model by a measure of the inharmonic parts of the signal, such as HNR (Garellek, 2019), the interpretation of H1-H2 becomes more meaningful. In our case, HNR increases from unaccented to accented supporting the conclusion of a tendency away from creaky voice toward the more modal voice. In the differentiation of accented focus types (broad, narrow, and contrastive focus) we do not obtain clear results for voice quality. In broad vs. contrastive focus, narrow vs. contrastive focus (vowel /a/), and broad vs. narrow focus (vowel /a/), we find increased H1^*^-H2^*^ values, but the results of HNR do not provide clear evidence for voice quality changes in any of these cases. To conclude, voice quality seems to play a role in accentuation but not in the gradation of prosodic prominence beyond accent.

All these results show that focus, similar to many other phonetic phenomena (Gafos et al., 2019; e.g., Kingston and Diehl, 1994), is realized via multiple different cues. These findings mesh well with new perception studies on prominence which have shown that several of these phonetic variables matter (Cole et al., 2010; Baumann and Winter, 2018; Bishop et al., 2020). Winter (2014) and others have proposed that this feature of phonetic systems—realization via multiple cues—increases the robustness of speech communication, i.e., the availability of different cues allows retrieving prosody-related meaning even if particular cues are not available by context. It is also known that individual listeners weigh different cues differently, as has been demonstrated for prominence by Baumann and Winter (2018). The availability of multiple cues for the same linguistic contrast thus also safeguards prominence perception against individual differences in perception. The multiplicity of phonetic variables in the expression of pragmatic meaning may not only enhance robustness in speech perception but also have the potential to enhance flexibility in speech production. On the one hand, future research needs to take into account how different speakers or groups of speakers use the parameters (e.g., by looking at the random effects of our models more carefully). On the other hand, it may be interesting to see how the phonetic variables are weighted in certain contexts, for example under adverse communication conditions that can be either language-internal (e.g., short vowels, small portions of voicing, see Niebuhr, 2012; Ritter and Roettger, 2014) or language-external (e.g., noise, less optimal hearing conditions).

## Data Availability Statement

Publicly available datasets were analyzed in this study. This data can be found here: https://osf.io/92ay8.

## Ethics Statement

The studies involving human participants were reviewed and approved by the Ethics Committee of the University of Cologne. The participants provided their written informed consent to participate in this study.

## Author Contributions

SR and DM contributed to the design and elicitation of the data set. BW, SR, and DM contributed to the conception of the statistical analysis. SR and BW contributed to the realization and coding of the statistical analysis. SR drafted the manuscript. All authors contributed to manuscript revision, read, and approved the submitted version.

## Funding

This work was supported by the German Research Foundation (DFG) as part of the SFB1252 Prominence in Language (Project-ID 281511265), project A04 Dynamic modeling of prosodic prominence at the University of Cologne. BW was supported by the UKRI Future Leaders Fellowship MR/T040505/1.

## Conflict of Interest

The authors declare that the research was conducted in the absence of any commercial or financial relationships that could be construed as a potential conflict of interest.

## Publisher's Note

All claims expressed in this article are solely those of the authors and do not necessarily represent those of their affiliated organizations, or those of the publisher, the editors and the reviewers. Any product that may be evaluated in this article, or claim that may be made by its manufacturer, is not guaranteed or endorsed by the publisher.

## Footnotes

^1^This applies to tonal onglide in the version used by Grice et al. (2017). In the present article, it is operationalized slightly differently.

^2^Similar to the classic studies of Fry (1955) and Lieberman (1960), and Kent and Netsell (1971) intended to examine the influence of stress on jaw and tongue kinematics by using stress minimal pairs of verbs and nouns in English (*im'pact* vs. '*impact*). The way the speech material is designed makes it very likely that all stressed syllables also received the nuclear pitch accent.

^3^The same data are also reported in Roessig et al. (2019), Roessig and Mücke (2019) and Roessig (2021) with a subset of the measure presented here.

^4^The illustrations of the questions in thought bubbles were as follows: The tool in question was shown on top of the target object in the case of background and contrastive focus; a simple question mark was shown in the case of broad focus; the target object and the question word “wo?” (“where?”) were shown with a question mark in the case of narrow focus.

^5^The measure of the vocalic tongue gesture duration may be influenced by the consonantal context of the vowel. However, this influence is controlled for by our study design: First, our data set is balanced for the consonants across focus conditions, i.e., in all four focus conditions, the same target words with the same consonants were used. Second, our analysis uses target word as a random effect (intercept and slope). Third, we use the backmost tongue sensor that has the weakest influence of tongue tip movements.

^6^Posterior predictive checks revealed that for two phonetic variables, tonal onglide and peak alignment, our models could not generate distributions that deal with the bimodality that was present in the data. The appropriate way to model these variables would be a mixture model as used in Roessig (2021). However, since we wanted to keep the model designs exactly the same for all variables to facilitate comparability, and because mixture models seemed inappropriate for all other variables, we decided to accept the deviation in the fits. As discussed in the results section, the respective variables still yield the largest effect sizes.
